# TriHex Technology and Reversal of Solar Elastosis: Dispelling Some Myths

**DOI:** 10.1111/jocd.70824

**Published:** 2026-03-31

**Authors:** Alan D. Widgerow, Karl Napekoski, Faiza Shafiq, Tiffany Robison

**Affiliations:** ^1^ Division Chief Research, Professor Plastic Surgery, Center for Tissue Engineering University of California Irvine US; ^2^ Alastin Skincare, Inc. a Galderma Company US; ^3^ Dermatopathologist Midwest Diagnostic Pathology, Department of Pathology Wake Forest University School of Medicine US

## Abstract

**Background:**

Solar elastosis (SE) is the end result of long‐term sun damage, characterized by a buildup of dystrophic material in the extracellular matrix (ECM) surrounded by fragmented collagen and dysfunctional, aggregated elastin. This degenerative process was once thought to be irreversible but is now recognized as dynamic, with the potential for regenerative change through appropriate interventions. New stains also enable the detection of newly formed elastin and collagen as part of this reversal process.

**Materials and Methods:**

A retrospective analysis of multiple clinical studies (624 patients, 80 biopsies, 31 reported solar elastosis) was conducted, focusing on the use of TriHex and TriHex+ peptide‐based technology (Alastin Skincare Inc., Carlsbad, CA). Biopsies were evaluated using novel histologic stains, including Herovici and Movat, alongside conventional Hematoxylin & Eosin (H&E) and CD44. Markers such as Collagen IV and Laminin at the dermo‐epidermal junction (DEJ) were also assessed to identify evidence of regenerative reversal of solar elastosis, ECM remodeling, and new elastin formation.

**Results:**

The regenerative reversal of solar elastosis was observed across multiple clinical trials. The TriHex formulations consistently produced significant improvements in ECM structure. Reported histology in 74% of cases showed clear removal of amorphous elastotic deposits and restoration of the ECM, including new elastin fiber formation, strengthening of the DEJ with increased Collagen IV and Laminin, new rete ridge architecture, and positive hydration effects evidenced by increased CD44 staining.

**Conclusion:**

This review positively challenges a long‐standing myth and clearly establishes a new perspective: solar elastosis is reversible. The peptide‐based TriHex Technology can remove the elastotic amorphous material from the ECM and rebuild it with new elastin and collagen fibers. Clinically and histologically, this shows that chronic sun damage can be repaired in the dermis, supporting a shift from superficial treatments to a more targeted regenerative approach focused on restoring the structural integrity of the skin.

## 1 Background

Solar elastosis is characterized by an accumulation of abnormal or dystrophic elastin in the dermis caused by prolonged ultraviolet (UV) light exposure and the resulting photodamage. This typically results from extracellular matrix (ECM) breakdown and thinning, with fragmentation of collagen and elastin fibers, which may have undergone alternative mRNA splicing [1].

Unlike normal intrinsic aging, which involves thinning of the ECM and the breakdown and loss of elastin fibers, UV exposure results in an accumulation of dystrophic elastotic material within the reticular dermis. This accumulated elastin is mainly composed of tropoelastin, the core protein of elastin fibers, while fibrillin, fibulin, and the delicate vertical fibers that normally extend to the dermo‐epidermal junction (DEJ) in candelabra‐like patterns are the first components of elastin to be damaged when affected by UV light [1, 2, 3].

Having outlined these changes, the question still remained: Can solar elastosis be reversed, or is it a degenerative endpoint as early researchers believed? Why did this perspective persist for so long? Several reasons seem to contribute—conventional stains were unable to differentiate between new and degraded elastin [2]. Few topical treatments targeted ECM remodeling, focusing more on epidermal and surface changes rather than dermal alterations. Similarly, clinical improvements were often judged superficially without the regenerative focus we have today.

This paper adds to the growing evidence that solar elastosis can indeed be reversed, in this case using peptide‐based technology, and demonstrating this reversal using carefully validated histological data with novel staining techniques. Additionally, the objective of this paper is also to demonstrate the regenerative capacity of the peptide based TriHex Technology to modulate the ECM and restore the structural integrity of photodamaged skin. This was achieved utilizing a retrospective review of histology from previously published histology data including novel specialized staining techniques.

TriHex Technology is a peptide‐based ECM remodeling platform composed of a tripeptide (Gly‐His‐Lys), hexapeptide, and supporting actives. These peptides recycle the sun‐damaged, dysfunctional ECM by removing fragmented collagen and elastin and replacing them with newly formed collagen and elastin [4, 5, 6, 7].

## 2 Materials and Methods

We examined the histology results of publications, pooled data and trials conducted with various products based on TriHex Technology [2, 5, 6, 7, 8, 9, 10, 11, 12, 13] (between 2017 to 2025). These publications are part of the repository of publications on TriHex Technology formulations that have been published in various peer review journals. The publications were searched using key words such as “solar elastosis” along with reviewing all biopsy stains and histological changes that were depicting reversal of SE were also selected for analysis and discussion. A total of 624 patients had been evaluated with various formulations containing TriHex Technology across all these publications. Of these patients, 80 participants underwent biopsies on various anatomical locations of the body treated with formulations containing TriHex Technology. Out of these 80 patients, 31 were reported as having SE changes histologically. Histological changes in these publications were evaluated by an independent dermatopathologist using the following stains: Hematoxylin & Eosin (H&E) and Versican. Importantly, the histology data included in this review were derived from treatment with TriHex Technology, without any adjunctive procedural intervention. Overall, clinical trials, evidence of reversal of SE, and representative examples demonstrating definitive reversal are presented.

## 3 Results

Over the past 10 years, research using peptide technologies (especially TriHex Technology) and active agents has provided valuable data on changes in the ECM and epidermal layers after topical application. Specifically, focusing on changes related to solar elastosis as described above, we documented histological changes that clearly showed the reversal of solar elastosis in multiple publications. We examined clinical studies, pooled data and publications between 2017 to 2025 involving 80 patients' histology, of whom 31 had been specifically evaluated for SE‐related outcomes. Among these 31 individuals, 23 were reported as showing histological evidence of SE reversal, corresponding to a 74% rate of reported reversal. This high proportion of responders shows a clinically meaningful and significant shift from the long‐standing paradigm that SE is largely irreversible with topical therapy. Some of the representative histological findings are summarized below, further illustrating the consistent pattern of ECM remodeling, restoration and SE reversal, across these investigations.

### 3.1 The New Paradigm: Solar Elastosis Reversal Is Now Possible

The past few years have seen a significant shift toward regenerative changes within the deeper layers of the skin. Notably, the appearance of early regenerative elastin and collagen fibers, identified through specialized staining techniques, has provided the necessary evidence that remodeling of the ECM with substantial density changes, the disappearance of voids or cystic collections, and their replacement with neocollagenesis and neoelastogenesis represent a reversal of solar elastosis.

### 3.2 Evidence

#### 3.2.1 Staining Profiles/Histologic Evidence

**FIGURE 1:**
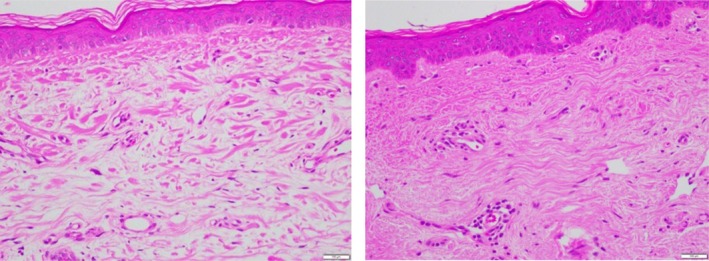
Baseline pre‐treatment (left) and 3 weeks post topical treatment with TriHex technology formulation (right).

To be more specific, each staining profile provides different levels of supporting evidence of these regenerative changes:

1. Hematoxylin & Eosin (H&E) (Figure 1).

Figure 1: The H&E‐stained slides above, captured in a clinical study using TriHex+ Technology (Alastin Skincare Inc., Carlsbad, CA) (in submission [12]) beautifully demonstrate several features related to SE and its reversal. In the baseline slide before topical application (left), multiple voids of homogeneous dystrophic material are evident in the ECM, with sparse collagen fibers, and the entire matrix appears diluted and sparse. Additionally, there is effacement of the DEJ, possibly including dissolution and fragmentation of the border, along with the deposition of degraded DEJ material just below the junction. The basal stem cells are reduced in number and sparsely distributed. After just 3 weeks of topical application, dramatic changes are visible (right). The ECM is now filled with densely packed collagen fibers, voids are significantly decreased, and the new tissue seems to be “pushing” down the elastotic material into the reticular dermis, with regenerative changes occurring in the papillary dermis and progress downward. The DEJ is much better defined, showing early rete pegs formation and reduced effacement. Basal stem cells are more plentiful and better spaced. Overall, this presents a very regenerative picture, and clearly demonstrates a reversal of degenerative changes. Applicable study reference [14].

**FIGURE 2:**
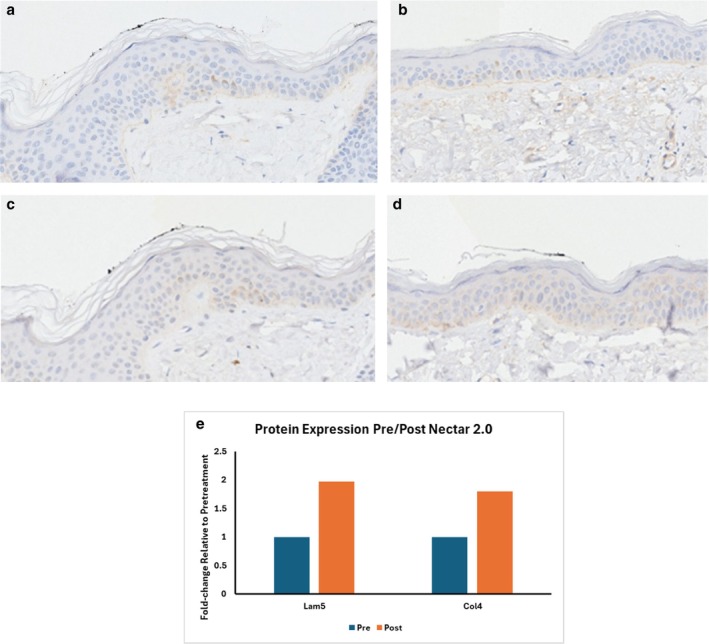
(a) Col IV pre‐treatment. (b) Col IV 3 weeks post treatment with TriHex technology formulation. (c) Laminin pre‐treatment. (d) Laminin 3 weeks post treatment with TriHex Technology formulation. (e) Showing fold‐change increase in both Lam5 and Col4 from pre‐treatment to post‐treatment with Nectar 2.0.

2 DEJ—Collagen IV Figure 2a,b and Laminin Figure 2c,d.

The DEJ is also impacted by SE—chronic UV exposure weakens and reduces the mechanical connection between the epidermis and the dermis, making the skin more vulnerable to mechanical stresses. This occurs due to a loss of structural protein support such as Collagen IV and laminins. A typical response to topical treatments is the subtle but clear highlighting of the DEJ by Collagen IV, as shown in Figure 2a, displays intermittent staining along the DEJ border; 3 weeks later, a continuous line of Collagen IV appears (Figure 2b), indicating reconstitution of the DEJ. The reactive oxygen species (ROS) generate damage to proteins like laminin and Collagen IV in the zone of the basement membrane, disrupting skin regeneration and repair [15]. The staining patterns are different, where Collagen IV demonstrates a typical linear staining pattern, while laminin exhibits a more complex network style distribution, as seen in Figure 2c and Figure 2d (increased density of staining after treatment—seen in 2d). Applicable study reference [14].

Quantitative image analysis of the histology revealed that Lam5 intensity rose from an average of 0.647 pre‐treatment to 1.276 post‐treatment with Nectar 2.0, corresponding to nearly two fold increase (fold‐change≈1.97). Similarly, ColIV exhibited an increase from an average of 1.86 (pre‐treatment) to 3.35 (post‐treatment with Nectar 2.0), yielding a fold‐change of about 1.8. These findings indicates that treatment with Nectar 2.0 significantly upregulated these ECM components. These enhanced expressions suggests the improvement in structural integrity and role of Nectar 2.0 in promoting matrix remodeling and healthier DEJ (Figure 2e).

**FIGURE 3:**
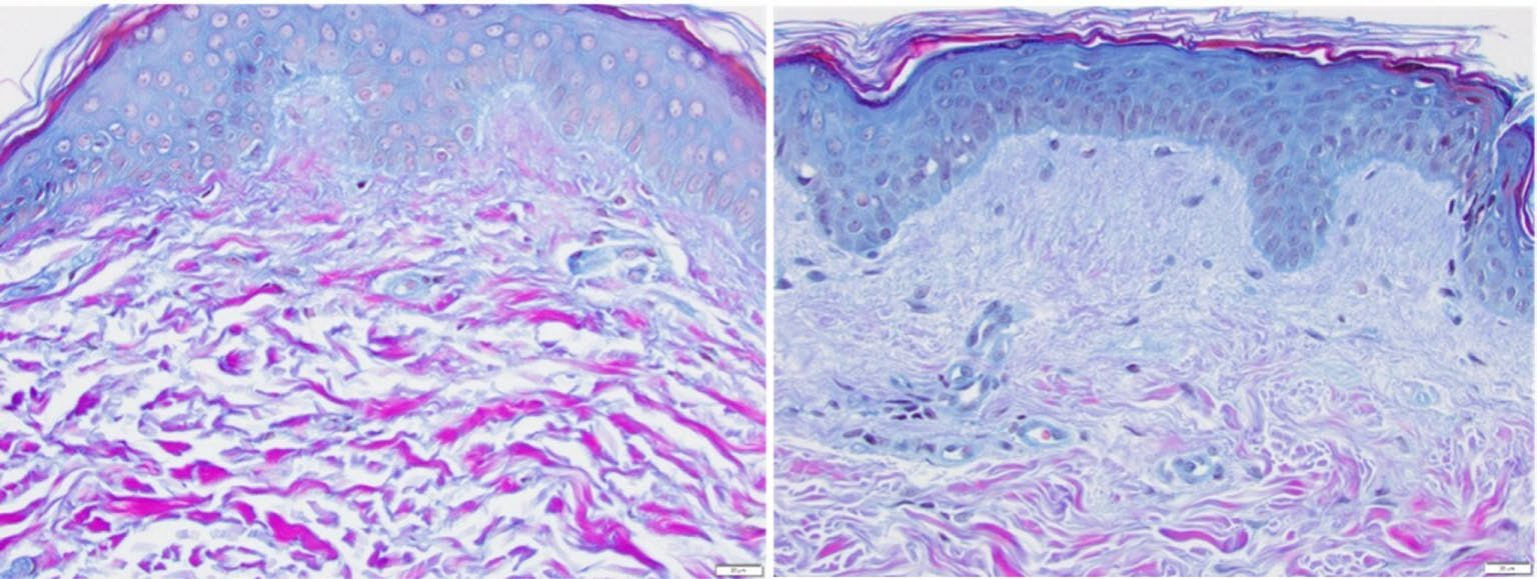
Herovici stain demonstrating magenta to blue color conversion representing new early collagen fiber formation (represented by mucopolysaccharides).

3 Herovici (Figure 3). Baseline pre‐treatment (left) and 3 months post topical treatment (right)

The Herovici stain is particularly useful for identifying neocollagenesis, detecting new immature collagen fiber formation by the presence of mucopolysaccharides that collect and precede the collagen fiber formation. Following treatment, new fine fibers are evidenced by the blue staining. There is a marked change in the ratio of new fibers (blue) to aged collagen fibers (magenta color). This remarkable increase in new fine collagen fibers represents regenerative collagenesis. This is evident in the slides below, where the change in ECM from an SE picture (left) to a new regenerative one is apparent 3 months post topical treatment with TriHex Technology formulation (right). Applicable study reference [16].

**FIGURE 4:**
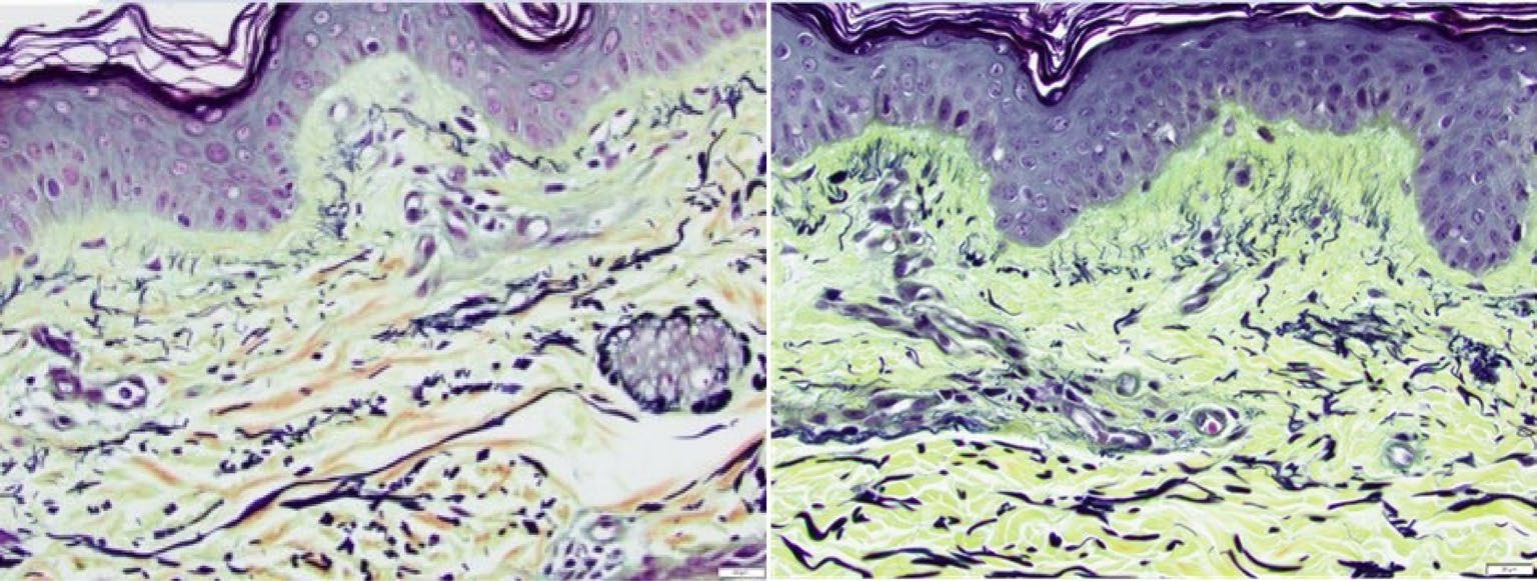
Movat stain demonstrating fine early fiber formation in papillary dermis on the right depicting neoelastogenesis.

4 Movat (Figure 4). Baseline pre‐treatment (left) and 3 months post topical treatment with TriHex Technology formulation (right)

The Movat stain, similar to Herovici, demonstrates early fiber formation, but this time related to elastin fibers. In particular, close attention should be paid to the papillary dermis where fine black fibers running perpendicular to the DEJ are seen, representing neoelastogenesis, and the replacement mainly of fibrillin, the first component of elastin to be affected and replaced by UV damage and SE. In the slides below, not only are new fibers evident in the papillary dermis, but the amorphous material seen in the ECM prior to treatment (left) has been replaced by an improved ECM, and elastotic material has been replaced by regenerating new elastin fibers (right). Applicable study reference [16].

**FIGURE 5:**
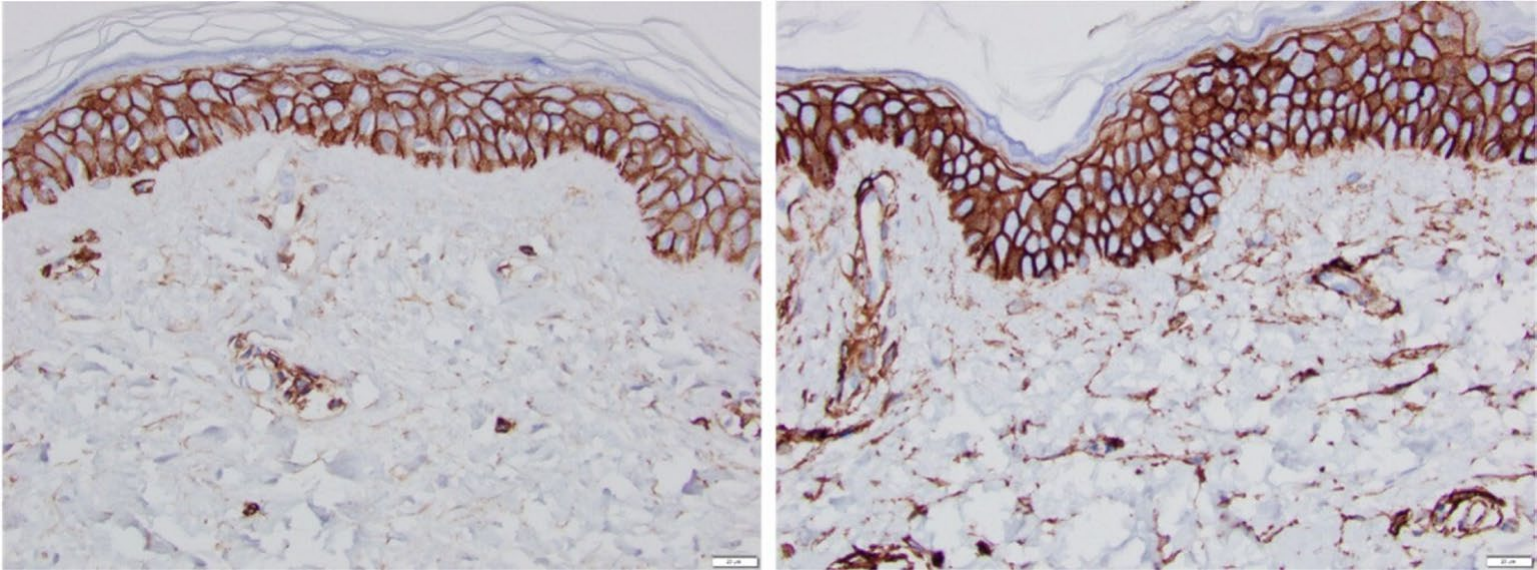
CD44 surface cell staining representing HA levels (note marked increase in intensity of staining on the right).

5 CD44 (Figure 5). Baseline pre‐treatment (left) and 3 months post topical treatment with TriHex technology formulation (right)

CD44 is a cellular surface marker present on many cells. In this context, we use CD44 to represent hyaluronic acid (HA) and hydration of epithelial and fibroblast cells. The intensity of staining (brown) correlates with HA stimulation, and the increase in intensity is clearly visible in Figure 5 at 3 months post treatment (right). This rise in HA levels is also associated with a reversal of SE and remodeling of the ECM. Applicable study reference [16].

### 3.3 TriHex Peptides

TriHex Technology comprises a peptide blend selected for its ability to modulate the ECM. Through a series of experiments and biopsies, it was shown that these peptides gradually break down clumped collagen and elastin fragments, then encourage replacement with new collagen and elastin, effectively recycling the ECM. This concept has been applied not only to maintain skin health and reverse photodamage but also to address waste product accumulation and changes associated with SE [2, 5, 6, 7, 11].

### 3.4 Clinical Trials (Topicals Alone)

For this review, clinical publications from 2015 to 2025 were assessed to gather evidence for the reversal of SE [2, 4, 5, 6, 7, 8, 9, 10, 11, 12]. Clearly, the histological results described above provide the strongest evidence for this reversal, but examining changes related to skin health offers the most direct visible evidence of sun damage reversal. Therefore, the reports of skin health benefits—including significant changes in skin volume, elasticity, hydration and radiance, which are documented across these studies—are relevant to the clinical effects of SE reversal.

### 3.5 Energy‐Based Synergy With Topicals

Lastly, as with many skin conditions, combining device or injectable treatments with topical therapy often provides synergistic benefits. These combinations have not been examined in detail in this review, as they increase the variables and make it harder to determine which modality contributes most to the changes in SE. However, it should be noted that laser/RF/microneedling promotes repair when paired with regenerative topicals, making it a logical combination—the device being the booster and the topical the maintenance.

## 4 Discussion

Sun exposed skin is subjected to daily photodamage that cumulatively results in fragmentation of collagen and elastin and additional disorganization of the extracellular matrix (ECM) of the dermis. In addition, aging and “wear and tear” add to this damage with the changes in skin manifesting as loss of elasticity, fine lines, pigmentation changes and senescence of cells like the fibroblasts [17].

TriHex Technology is a peptide‐based ECM remodeling platform composed of a tripeptide (Gly‐His‐Lys), hexapeptide, and supporting actives. These peptides support clearance of fragmented collagen/elastin via signaling and stimulating fibroblasts to increase production of new collagen, elastin, and glycosaminoglycans, effectively recycling dysfunctional ECM [4, 5, 6, 7]. In 2025, TriHex technology was advanced, incorporating a proprietary, patent‐pending Octapeptide‐45(Octa) into the TriHex technology with added benefits on DEJ, hydration, fibroblast senescence, and elastin formation [18]. The analysis carried out in this paper includes both the original formula and the advanced formula, both renditions demonstrating reversal of solar elastosis. Traditional immunohistochemistry (IHC) staining has failed to distinguish degenerated elastin from new elastin fibers produced through various topical strategies. Therefore, our group introduced new staining techniques to differentiate old elastin (solar elastotic) from newly formed elastin [2]. This is itself a relatively new concept, as between the 1980s and 2000s, where many authors described elastotic changes as degenerative endpoints with little possibility of reversal and regeneration [19, 20, 21].

To summarize current understanding, elastic fibers are made up of an amorphous tropoelastin core surrounded by multiple microfibrils. The tropoelastin core is cross‐linked during assembly with the aid of a scaffold produced by microfibrils, including fibrillin 1 and 2, fibulin 5, microfibril‐associated glycoproteins (MAGPs), and latent transforming growth factor β‐binding proteins (LTBPs) [1]. Solar elastosis involves the UV‐induced buildup of elastotic material in the dermis. This process includes the loss of LTBP‐4 and abnormal accumulation of elastin, Versican (which is damaged by UV light and loses its hyaluronic acid (HA) binding capacity), hyaluronic acid, fibrillin, fibulin‐2, and fibulin‐5, all aggregated in a disorganized mass that we recognize as solar elastosis [22, 23, 24, 25].

Investigators first identified areas containing dense dystrophic elastin fibers mixed with void spaces early on. These locular or multilocular cystic spaces appeared either empty or filled with finely fibrillar or granular material, particularly in individuals ages 50–70 years. However, changes were already visible in the 30–50 years age range, with cysts or lacunae present from that age [26]. In an attempt to induce these changes in the ECM and elastin, investigators used digestive enzymes such as elastase and chymotrypsin and were able to produce degeneration of elastic fibers similar to that seen in UV‐damaged skin [26].

The belief at the time was that normal elastin synthesis no longer occurred after age 20, but new abnormal fibers began to form between 20 and 40 years due to photodamage secondary to prolonged UV light exposure.

Early on, Kligman distinguished age‐related changes from those caused by UV light by describing the thickening, curling, and branching of sun‐damaged elastin fibers, which gradually transformed into amorphous masses within the dermis [14]. What could have caused this? Since elastin accounts for only 2%–4% of the dermis, other proteins like fibrillin, glycosaminoglycans—such as hyaluronic acid (HA) and Versican—and lysozymes, as suggested by Bernstein et al., must also be involved in this transformation [20, 27].

As the understanding evolved, new cellular participants were considered in the process. In particular, while chronic sun damage promotes the release of collagen‐destroying enzymes (MMP‐1) from keratinocytes and fibroblasts, acute UV light exposure leads to an influx of neutrophils into the dermis. These neutrophils are packed with proteolytic enzymes, with neutrophil elastase having a specific effect on elastin fiber degradation [21, 28].

As demonstrated here, TriHex Technology (Alastin Skincare Inc., Carlsbad, CA) uniquely reverses solar elastosis by first clearing damaged ECM and then actively stimulating the production of new, functional dermal components.

The primary limitations of this analysis include its retrospective design and reliance on pooled histological data from patients across multiple previously published studies rather than a single, prospectively designed investigation focused specifically on solar elastosis. The inclusion of biopsy samples collected from various anatomical sites introduces heterogeneity in baseline photodamage, although it also provides a broader view of tissue response across different levels of sun exposure. Additionally, treatment durations varied among the included studies, which may complicate direct comparison of outcomes but simultaneously offers insight into both early and longer term regenerative effects associated with TriHex Technology. Collectively, while these limitations should be considered when interpreting the findings, the consistent pattern of extracellular matrix remodeling and reversal of solar elastosis observed across studies strengthens the overall conclusions.

Future research objectives include exploring solar elastosis reversal in the context of combination treatments with energy‐based devices. Although formulations containing TriHex Technology are already routinely paired with lasers, microneedling, and other device‐driven procedures in clinical practice, and it has been well established that TriHex Technology improves ECM remodeling, enhances treatment outcomes, and reduces procedural downtime, the specific impact of these combination approaches on solar elastosis reversal has not yet been systematically investigated. Given the established histological evidence that TriHex Technology alone can clear elastotic material and stimulate new collagen and elastin deposition, evaluating its impact when used alongside energy‐based devices will be critical for determining whether these modalities act additively or synergistically to further enhance dermal regeneration and accelerate reversal of solar elastosis.

## 5 Conclusion

This review positively challenges a long‐standing myth and clearly presents a new perspective: solar elastosis is reversible. The peptide‐based technology can signal to remove the elastotic amorphous material from the ECM and rebuild it with new elastin and collagen fibers. Clinically and histologically, this demonstrates that chronic sun damage can be repaired in the dermis, supporting a shift from superficial treatments to a more targeted regenerative approach focused on restoring the skin's structural integrity.

## Author Contributions

A.D.W. – developed the science, analysis, paper writing, K.N. – Histology, paper writing, F.S. – paper writing, T.R. – paper writing.

## Funding

Funding applied for these studies was from Alastin, a Galderma company. This work was supported by Alastin Skincare Inc., a Galderma company.

## Ethics Statement

Ethical guidelines for Wiley were followed and IRB‐informed consent was obtained in all studies referred to in this publication.

## Conflicts of Interest

Several authors (A.D.W., F.S., T.R.) are employees of Alastin Skincare. K.N. serves as a consultant Dermatopathologist. All clinical trials included in this retrospective review were funded by Alastin Skincare, A Galderma Company.

## Data Availability

The data that support the findings of this study are available from the corresponding author upon reasonable request.
